# Hemorrhage from the Lungs*A paper read before the Oneida County Medical Society, October 14, 1879.

**Published:** 1880-01

**Authors:** Willis E. Ford

**Affiliations:** Utica, N. Y.


### HEMORRHAGES FROM THE LUNGS.*

BY WILLIS E. FORD, UTICA, N. Y.

MEDICAL MEN have always differed widely in their opinions as to the significance of hemorrhage from the lungs. Sylvius, Sydenham, Boerhaave and Andral held the doctrine that hemorrhage caused the diseases of lungs which were observed to follow in so many instances. Laennec taught that hemorrhage was only one of the symptoms of already existing disease of the lungs. It is interesting to note that many careful observers of disease have now come back to something akin to the old views of pulmonary hemorrhage and regard it as not always the result of phthisis but as an accident which in certain cases may be harmless, while in other cases it may bring about destructive disease of the lungs. Niemeyer and his followers are foremost in advocating this theory, and of course this must be the tendency of all those who believe in the doctrine of the local origin of phthisis. In judging of medical facts, it is sometimes unfortunate to have a theory too vividly in mind, for the judgment may be obscured regarding the individual case, and I believe this has been true regarding pulmonary hemorrhages. If, on the one hand, they are looked upon as harmless, or even beneficial in relieving a congested lung, then nothing is done and the patient may drift rapidly into hopeless disease. On the other hand, if the hemorrhages are looked upon as the simple announcements of existing consumption, then the case is thought to be hopeless and a routine treatment is applied.

*A paper read before the Oneida County Medical Society, October 14, 1879.

I believe that pulmonary hemorrhages have such a variety of causes, and the ultimate results of such hemorrhages are so widely different, that each case requires the most careful examination and study, and that there can be no routine treatment.

Hemorrhages, occurring during the progress of an ordinary course of phthisis, are due to the giving way of the coats of some blood-vessel at the site of the trouble. They are usually seen early in the formation of an abscess, and their situation can be easily made out. This form of hemorrhage need not be dwelt upon here, neither the hemorrhages caused by traumatism.

The most important class of cases, and that to which I wish especially to call attention in this paper, comprises those who have a hemorrhage from the lungs, without knowing that they have had any special trouble there before. Now, to assume that all such persons, even though they may have a family history which predisposes them to consumption, have already a tubercular deposit in the lungs and are therefore doomed—is, I believe, unfair, and is apt to cut such patients off from appropriate treatment. Following out the history of these persons, we find many do recover fully, and that without any special treatment, though many others gradually sink into fatal disease of the lungs. A careful examination of the facts after such a hemorrhage will in most instances show that there has been a gradual decline in general health for some time, usually for several months. There has been a loss of flesh and strength, a falling off of the appetite, and an inability to do easily the customary work. These symptoms may pass unnoticed, and even if pain occurred, it would not be heeded by many persons, or remembered till after the hemorrhage. Of course, immediately after this, there is great pallor and weakness, due partly to the shock and the apprehension, and partly to actual loss of blood. If an examination of the lung be made now, in a large number of cases no consolidation will be found, and the air will enter fully all parts of the lung, and, if the hemorrhage has been stopped for some hours, it will be difficult to detect the spot from which the blood came. Upon listening to the breathing, however, there will be a harshness of respiration over certain portions of the lung, and the attentive and practiced ear will discover that this harshness is made up of innumerable little rales that are soft and moist. These crepitant and sub-crepitant rales are heard from day to day in the same place, and are not removed by coughing. At the same time the air will be heard freely entering the lung beneath them.

A case illustrating this condition, and which I observed carefully during its entire progress, was a man aged thirty-three years, who, from close confinement and hard work, ran down in health for a month or two. He lost flesh and strength, but was not aware of any lung trouble, until in getting up one morning he began to spit blood. The hemorrhage was not a copious one, but when I saw him a few hours later, he was much prostrated, his temperature was raised a degree, and his pulse was a hundred and ten. There was evidence of plastic exudation over a small part of the left lung. He got about again in a few days, but he had pleuritic adhesions where the original trouble began; these adhesions became organized; an appreciable consolidation of lung took place at this point, and for six months or more he had cough, profuse expectoration, and at times night sweats. He was a judicious man, however, followed carefully his treatment, and resolution slowly took place. He has remained well for two or three years. Another case parallel in its early stage was seen last year in a young woman about twenty-one years of age, unmarried, and a teacher by occupation. During a term of school when her work was hard her mother died. The grief and anxiety regarding her family, and her hard work, reduced her in strength, and after a few weeks she had a profuse hemorrhage from the lungs. I saw her a year later, and during this interval she had experienced pain in the side, had persistent cough and profuse expectoration. At the apex of the left lung there was found an extensive pleuritic adhesion with some consolidation beneath it, though air could be heard to enter the lung freely underneath. She was then subject to frequent hemorrhages, and night sweats, loss of appetite and hectic. She steadily failed, and recently died. Another case: a man for whom I prescribed once or twice during the summer for want of strength to pursue his ordinary vocations, and loss of appetite; he had no symptoms of lung disease, but was simply depressed by overwork and anxiety; a few weeks ago while away from home and very busy, he suddenly began to spit blood; it was bright red blood, came without any effort of coughing, and was quite profuse; this continued for two or three days, and was accompanied by pain in the left side. His physician found plastic exudation of the pleura, covering the lower lobe of the left lung; and speedily he had pneumonia of this lobe which ran through the ordinary stages. When he was supposed to be convalescing, he was one morning attacked by pain in the opposite side, and very soon began to spit blood as freely as before. An examination showed pleuritic exudation over the middle and lower lobes of the right side, and this was soon followed by consolidation of the lung underneath, and the usual course of pneumonia followed, excepting these two notable features. His respirations were never over eighteen per minute, and he continued to spit bright arterial blood until resolution was nearly completed.

Such cases as these, of which many more typical examples might be given, seem to point to the pleura as the original cause of the trouble. As we should naturally suppose, inter-pleural plastic exudation, together with swelling of the membrane, must exercise more or less pressure upon the lung beneath, and especially so after adhesions have formed, and have begun to contract, according to the law which governs all new formations of this kind. The air cells are compressed, and the circulation of the capillaries of the lung interfered with. The obstruction to the circulation of the blood from the pulmonary artery is not attended with any serious result, but to obstruct or to retard the flow of blood through the nutrient arteries, is simply to dam up the blood in the bronchial arteries whence they are derived. These nutrient arteries differ from all other arteries in the body, in that they have no venae comites. The blood which they carry to the tissues of the true respiratory system for their nutrition, is oxygenated as it passes along towards the pulmonary vein, never becoming venous in character. This back pressure explains the tendency to bronchorrhagia and bronchorrhœa in many cases, and we should endeavor in treatment to remove the obstruction which causes the oozing of blood or serum through the bronchial membranes. The treatment of the mucous membranes themselves, which are not at fault, is very naturally attended with no very flattering results. Any one, at all familiar with autopsies, will have been struck by the frequency of the occurrence of the evidences of inter-pleural fibrination even where no disease of the lung had been known to exist during life. Any prolonged depression of the vital powers is sufficient to produce it, such as over-work, venereal or alcoholic excesses, et cetera.

When these adhesions, of which I have spoken, are thoroughly organized, and their strength and number are increased by a continuation of the pleuritic trouble, then the contraction is sufficient to impede the entrance of air into a considerable portion of lung, and the patient begins to stoop forward to breathe hurriedly, and degeneration of lung, with the ordinary wasting of phthisis, begins. Dr. J. R. Leaming, who has recently made a very valuable contribution to the study of phthisis, asserts that nine-tenths of all the cases of phthisis in this climate begin in the way I have just described. Of course, this assumes that many of the cases of chronic catarrhal-pneumonia are not strictly affections of the bronchial mucous membranes, but that they have their origin in the pleura, and that the profuse expectoration and the hemorrhage are due to obstruction of the nutrient arteries, as I have just described. This is undoubtedly true of many cases, and especially so where early hemorrhages occur, and no ulcerative process or other local injury can be found to account for it. A careful physical examination will throw much light on this question. The rales that are heard are minute, very fine, and seem to be very close to the surface, while beneath them can be heard the true respiratory murmur, entirely unaccompanied by bronchial sound. Of course all this cannot be determined at the time of the hemorrhage itself, but it is very important that this class of cases should be recognized as early as possible, for many prove amenable to treatment.

Hemorrhages occurring as a result of strain or of excessive muscular exercise are quite common, and though not immediately dangerous, are apt, I believe, to cause disease of the lungs. If during the hemorrhage, either as a result of coughing or of forced inspiration, any considerable amount of blood be drawn into the air cells, it may act like other foreign bodies in exciting inflammation of the lung tissue. I know that Hertz in Ziemssen's Cyclopædia distinctly states that bronchial hemorrhages never cause phthisis. He says the inspired blood could cause superficial irritation in the bronchioles and alveoli which might lead to caseous metamorphosis of their contents, but that this is sure to be absorbed. He quotes Buhl as saying catarrhal pneumonia, as distinguished from desquamative, never causes extensive destruction of lung tissue. This seems a specious argument, one which I would gladly believe if possible, and I have brought up this subject, as to the effects of pulmonary hemorrhage, in this place in order to relate a case of hemorrhage from excessive muscular exertion which seems to bear directly upon this point. An asylum attendant, aged about 23, strong and well, so far as known by himself, his family or the physician who had seen him daily for two years in the discharge of his duties, being very anxious to overtake and return a patient who was escaping, and who had the start of him, ran for an hour or more. He was stimulated to do his utmost by the desire to excel other attendants, and also by the fear that unless he succeeded, some blame might be attached to him. Almost immediately afterward he began to spit blood. The hemorrhage came from the left lung, upper lobe, and was quite profuse. Of course his respirations at the time were very labored, and he was in the most favorable condition to draw in and retain some of the blood. A dry asthmatic cough followed, and though he was under the most favorable hygienic conditions, a consolidation of the lung took place, and within a year he had begun to develop a cavity, and all the symptoms of a progressive phthisis were present. There was no hereditary taint and all the circumstances seem to point to the hemorrhage as the starting point of his consumption. Another case of hemorrhage which I saw, came from muscular strain in lifting, which did not eventuate in any serious disease of the lungs, probably from the fact that the blood came slowly and but little was retained. A case of purpura also, in which extensive and prolonged hemorrhage from the lungs occurred, was followed by a lobular pneumonic inflammation, and caseous degeneration of the lungs. In this case I had examined the lungs carefully some time before, and with negative results. Only last week I saw a case of consumption in its last stages which had its beginning in hemorrhage of the lungs, less than one year ago. At that time her physician said that the bleeding would do no harm, and that she might go on with her regular work in the factory. I have notes of many cases which, like the preceding ones, go to show that hemorrhages may result in the most fatal consequences, and I think the cases are few in which a person may be told that a hemorrhage will do no harm, and may therefore be allowed to pass without careful watching and treatment.

I have always doubted those cases reported to have been the result of vicarious menstruation, or of an effort of nature to prevent disease, or to restore a lung already affected.

In the treatment of hemoptyses, I believe that too much reliance has heretofore been placed upon the administration of astringents. It is true that many hemorrhages are self-limiting, and cease, whether astringents have been used or not, when the immediate pressure is removed. Where there is great relaxation of the walls of the blood-vessels, however, with continuous oozing of blood, the so-called hemastatics do but little good. Dry cups to the chest are of immense service. Five or ten may be applied at once, and repeated once or twice, if necessary. Next in importance is opium, given in such doses as to contract the pupils, to allay pain and nervousness, and to reduce the respirations to from fourteen to seventeen per minute, and this should be continued for several hours after all hemorrhage has ceased. Ergot is useful in connection with opium, for it undoubtedly assists in stimulating the vaso-motor nerves to give contractility to the arteries. Absolute rest must be enjoined in every case. Where there is any ulcerative process going on within the lung, and it is reasonable to suppose that the walls of a blood-vessel have given way, then ice to the chest, together with ergot and opium, will do best.

In all cases of profuse hemorrhage the patient should lie upon the sound side, pretty well over upon the face, and should avoid, as much as possible, the act of coughing, so that blood will neither settle backward into the air cells, nor be drawn in by forced inspiration.

Of course the after treatment in those cases in which the pleura is involved is of vastly more importance than the immediate relief of symptoms; rest to the lung, so far as possible, should be secured. Counter-irritation by means of iodine or dry cups should be applied every other day, together with the administration of tonics, and in some cases stimulants. Where there is much cough or expectoration I have found the cold infusion of wild-cherry bark, with chloride of ammonia, as recommended by Dr. Leaming, very useful, more useful indeed than in any other kind of cough. In every instance of hemoptysis from any cause, where there remains in the lung any indication of retained blood, the utmost caution should be used. Absolute freedom from work, anxiety or annoyance must, if possible, be secured, and the patient must be given the best hygienic surroundings, while counter-irritation is kept up over the affected portion of the lung, and tonics are judiciously administered until there is complete restoration to health. I am convinced that there are no accidents so commonly befalling men in this latitude, deserving skillful investigation into their causation and more patient and persistent care afterward, and in which the welfare of the patient is more dependent upon good management, than in hemorrhage from the lungs.

